# Cognitive and motor aspects of cancer‐related fatigue

**DOI:** 10.1002/cam4.2490

**Published:** 2019-08-13

**Authors:** Li Rebekah Feng, Jeniece Regan, Joseph A. Shrader, Josephine Liwang, Alexander Ross, Saloni Kumar, Leorey N. Saligan

**Affiliations:** ^1^ National Institute of Nursing Research National Institutes of Health Bethesda Maryland; ^2^ Clinical Center Rehabilitation Medicine National Institutes of Health Bethesda Maryland

**Keywords:** cancer‐related fatigue, cognitive impairment, hand grip, physical fatigue, prostate cancer, radiation therapy, Stroop interference

## Abstract

**Background:**

Cancer‐related fatigue (CRF) is a debilitating symptom frequently reported by patients during and after treatment for cancer. CRF is a multidimensional experience and is often solely assessed by self‐report measures. The goal of the study is to examine the physical and cognitive aspects of self‐reported CRF using a cognitive function test and a physical fatigue index in order to provide objective measures that can characterize the CRF phenotype.

**Methods:**

A total of 59 subjects with nonmetastatic prostate cancer receiving external beam radiation therapy were included in the study. Fatigue was measured using the Functional Assessment of Cancer Therapy‐Fatigue (FACT‐F) questionnaire. Cognitive characteristics of CRF was measured using the Stroop Color‐Word Interference computerized test and the motor aspect of fatigue was measured using the static fatigue test using a handgrip dynamometer.

**Findings:**

Functional Assessment of Cancer Therapy‐Fatigue scores significantly correlated with the Stroop Interference score, but not performance accuracy in all test conditions. Fatigued subjects exhibited a more rapid decline to 50% of maximal strength and increased static fatigue index in the handgrip test, whereas maximal grip strength was not affected.

**Conclusions:**

The results suggest that CRF exhibits both cognitive and physical characteristics. Subjective fatigue was associated with increased time required to overcome cognitive interference, but not cognitive performance accuracy. Fatigued patients exhibited decreased physical endurance and the ability to sustain maximal strength over time. These objective measures may serve as valuable tools for clinicians to detect cognitive and physical impairment associated with CRF.

## Graphical abstract

We found that cancer‐related fatigue was associated with both increased cognitive interference and decreased physical endurance. This suggests that the overall sense of tiredness reported by cancer patients is both cognitive and physical in nature.

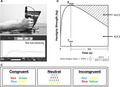

## 1 INTRODUCTION

The rate of death due to cancer overall has dropped considerably over the past two decades in the United States.^1^ As a result, there is increasing emphasis on long‐term side effects of cancer treatment and the quality of life of the growing cancer survivor population.^2^ Cancer‐related fatigue (CRF) is an extremely common and debilitating symptom reported by up to 80% of cancer patients during and after treatment completion.^3^ According to National Comprehensive Cancer Network (NCCN) guidelines, CRF is characterized by a persistent sense of physical, emotional, and cognitive tiredness, which is not proportional to recent activity and cannot be resolved by rest or sleep.^3^

Fatigue typically worsens during cancer treatment and can last up to years after treatment completion, severely impacting the patient's quality of life by reducing participation in normal daily activities.^3^, ^4^, ^5^ Accordingly, CRF has been identified as one of the top five first‐tier high‐priority research areas by the National Cancer Institute.^6^, ^7^ The diagnosis of CRF relies entirely on self‐reports.^8^ However, clinical consequences of CRF can be measured by objective measures; which will help advance our understanding of the underlying pathogenic mechanisms that might be targeted for optimal management.^9^ Fatigue in various disease conditions is thought to be multifactorial in nature and encompasses both physical and cognitive aspects.^10^ The overall sense of reduced energy could be a cognitive consequence of fatigue related to mental exhaustion, or physical fatigue presented as decreased motor performance over time.^11^ It is unclear whether these domains of CRF can be measured clinically through its cognitive or physical manifestations.^12^

Cognitive deficits are commonly observed in patients with cancer, particularly in complex information processing speed, working memory, learning efficiency, and executive functions.^13^, ^14^, ^15^ This illustrates the involvement of the fronto‐subcortical system which is consistent with the observation that lower cognitive performance and fatigue in cancer patients was accompanied by hyperactivation in the prefrontal cortex with increasing task difficulty.^13^, ^16^ Interestingly, self‐reported cognitive difficulties in cancer patients appeared to correlate with fatigue instead of decreased performance on neuropsychological tests.^17^ This suggests that CRF and cognitive impairment may be difficult to distinguish based solely on self‐report, and objective measures of cognitive performance are needed to characterize the true nature of CRF.

Physical/motor fatigue refers to the failure to maintain required force during sustained muscle contraction, commonly thought to be related to muscle tissue or the neuromuscular junction.^10^ Muscle weakness correlated with CRF both in patients with advanced cancer and cancer survivors.^18^, ^19^ However, complaints of physical fatigue by cancer patients tend to correlate with changes in normal daily activities and daytime sleepiness, while few studies utilized objective measures to characterize the subjective feeling of physical fatigue.^20^

To date, CRF has been examined in the literature based on self‐report, but additional investigations are warranted to fully understand the clinical manifestations of this debilitating symptom.^3^ Even though the sensation of fatigue has been correlated with both increased cognitive effort and physical functioning during daily activities,^21^, ^22^ few studies have examined the physical and cognitive aspects of fatigue in the same cohort. Our goal in this study is to examine the physical and cognitive aspects of self‐reported CRF using a cognitive function test and a physical fatigue index in order to provide objective measures that can characterize the CRF phenotype.

## 2 MATERIALS AND METHODS

### 2.1 Participants

The current study (NCT00852111) was approved in October 2008 by the Institutional Review Board of the National Institutes of Health (NIH), Bethesda, Maryland, USA. All subjects enrolled in this study were men, ≥18 years of age, diagnosed with nonmetastatic prostate cancer and scheduled to receive external beam radiation therapy. The entire radiation therapy (RT) lasted 38‐44 days, depending on the clinical stage of the prostate disease. The following were excluded from the study: (a) patients with chronic inflammatory disease, an unstable or end‐stage disease of any body system, a major psychiatric disorder within the past 5 years, any medical history of tuberculosis, any infectious disease such as HIV or hepatitis, or a second malignancy were excluded from the study; (b) those receiving chemotherapy or taking medications known to affect cytokine production, such as tranquilizers, steroids, and nonsteroidal anti‐inflammatory agents, were also excluded from this study. Subjects were recruited from April 2009 to January 2019 at the Magnuson Clinical Research Center at the National Institutes of Health. Signed written informed consents were obtained prior to study participation.

### 2.2 Instruments

Clinical data were obtained from chart review. Fatigue was measured using the 13‐item Functional Assessment of Cancer Therapy‐Fatigue (FACT‐F), which is a validated, reliable, stand‐alone measure of CRF (questionnaire items and scoring method can be found at http://www.facit.org).^23^ FACT‐F has demonstrated good internal consistency reliability with a Cronbach's alpha of 0.81 when tested in our study cohort. Total FACT‐F scores typically range from 16 to 53; lower scores indicate higher fatigue intensity. Clinically significant fatigue is defined using the standard Minimal Clinically Important Difference (MCID) method.^24^ Subjects were considered to have clinically meaningful RT‐induced fatigue when there was a decrease in ΔFACT‐F score of ≥3 points from baseline.^25^ The three‐point definition of ΔFACT‐F satisfies the minimally important difference threshold which has been shown to be clinically meaningful (defined by Cohen's 0.2 SD‐0.5 SD effect sizes).^26^, ^27^, ^28^

Depressive symptoms were measured using the Hamilton Depression Rating Scale (HAM‐D). High scores indicate severe depression: a score of 0‐7 indicates no depression, a score of 8‐16 indicates mild depression, and a score of ≥17 indicates moderate‐to‐severe depression.^29^ HAM‐D has demonstrated good internal consistency (standardized Cronbach's α = .67‐.80) and test‐retest reliability (Pearson correlation coefficient = 0.88, *P* < .001).^30^

### 2.3 Physical aspect of fatigue

**Figure 1:**
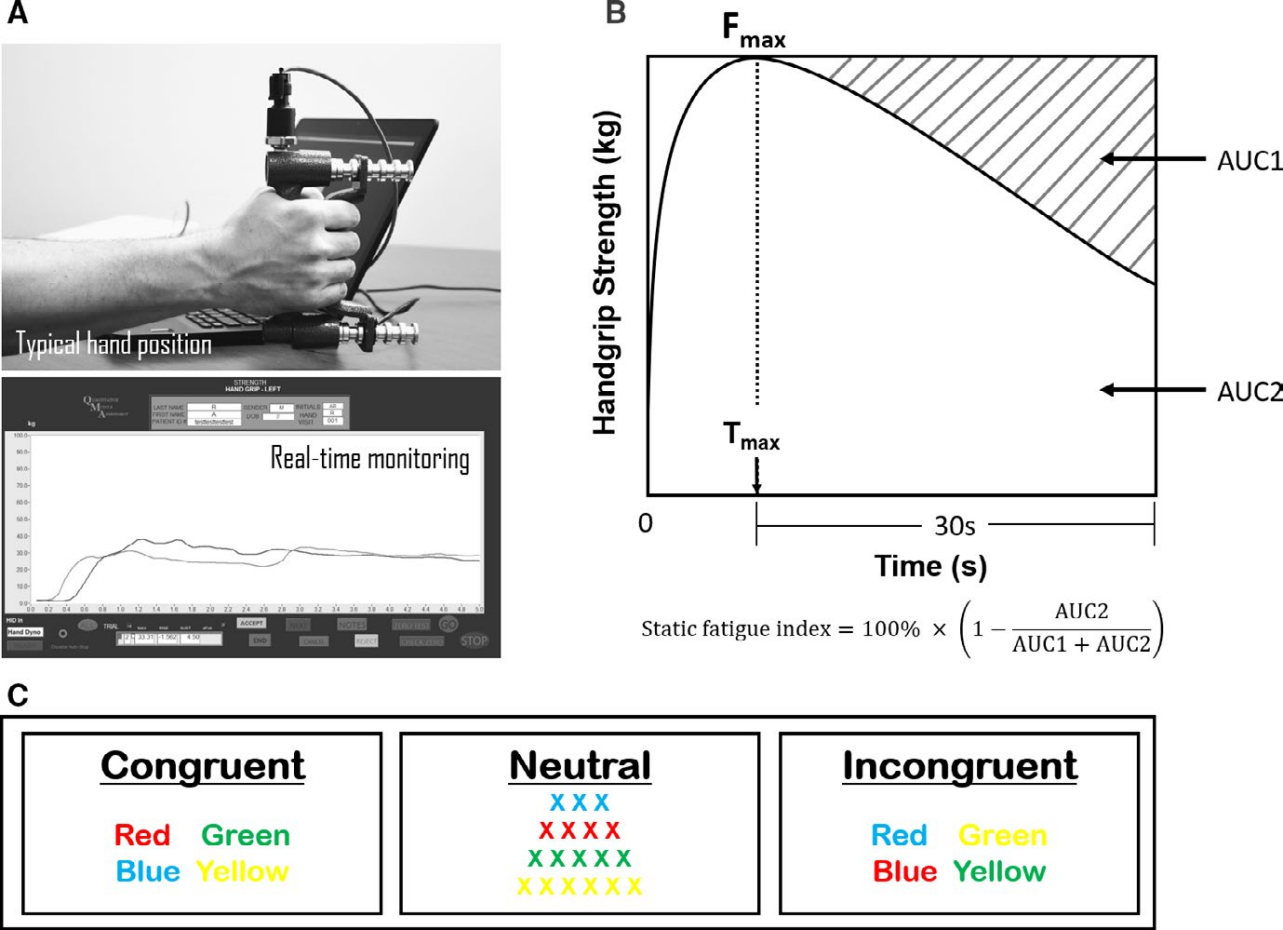
Hand grip measurement for assessing physical fatigue. A, Position of subject while performing Quantitative Muscle Assessment handgrip static fatigue test. Grip strength is monitored real‐time during the static fatigue test. B, Visual representation of static fatigue index calculation. *F*_max_: maximal hand grip strength during the static fatigue test. *T*_max_: time when *F*_max_ occurred. Static fatigue index is calculated as 100% × [1 − (AUC2/(AUC1 + AUC2))]. (C), Congruent, neutral, and incongruent conditions used in the Stroop Color‐Word Interference test

Patients underwent a primary static fatigue test with the use of a *Quantitative Muscle Assessment* (QMA) handheld dynamometer (Aeverl Medical, Gainesville, GA). QMA software version 4.2.1 was used to collect the time and force information (Aeverl Medical). The static hand grip test has shown high reliability for both time and work.^31^, ^32^ The subjects were seated in an upright position with the elbow flexed at 90° (Figure 1A, upper panel). Maximum hand grip strength test: two consecutive trials of maximum voluntary contraction of the nondominant hand was measured by requiring the subject to exert a maximum grip force for 5 seconds. Maximal Voluntary Isometric Contraction (MVIC) was calculated by averaging the two trials. After a 1‐minute interval, *the static fatigue test* was conducted by requiring subjects to maintain the maximum force for as long as possible. Hand grip strength was monitored real time during the static fatigue test (Figure 1A, lower panel) and the test was stopped when the strength dropped to 50% MVIC (Figure 1A). 50% Exhaustion Time (50% ET) was calculated as the time it took for maximal muscle contraction force to degrade to 50% of MVIC during static fatigue test.

The Static Fatigue Index (SFI) was calculated as previously described.^33^, ^34^ Briefly, the maximal hand grip strength was determined (*F*_max_) and SFI was calculated based on the time when *F*_max_ was reached (*T*_max_) to 30 seconds after *T*_max_. The shaded area in Figure 1B represents the SFI: the higher the SFI, the more *F*_max_ decreases over time. Hand grip strength‐time curve starting from *T*_max_ to 30 seconds post‐*T*_max_ was used to determine the area under the curve (AUC2). The hypothetical area under the curve is calculated as *F*_max_ multiplied by 30 seconds (AUC1 + AUC2). AUC1 is the difference between *F*_max_ multiplied by 30 seconds (AUC1 + AUC2) and the actual strength under the curve (AUC2) and represents fatigability. The SFI is calculated as such:

$$\text{Static Fatigue Index}\;\left(\text{SFI}\right)=100\%\times \left(1-\frac{\text{AUC}2}{\text{AUC}1+\text{AUC}2}\right)$$

### 2.4 Cognitive aspect of fatigue

The Stroop Color‐Word test is a well‐established tool designed to measure aspects of executive function, which is a cognitive domain associated with CRF in previous studies.^14^, ^35^, ^36^ Patients completed a computerized version of the Stroop Color‐Word Interference Test, in which colored computer keys were used to name colors. The Stroop test has shown good reliability and validity with a test‐retest correlation of 0.83‐0.91 and has been used in previous studies to test cognitive deficits in cancer patients.^37^, ^38^ The Stroop test included three types of stimuli: (a) neutral: patients named the color of 3‐6 character long strings of “X”s; (b) congruent: patients named the color of words in a congruent font color, eg, the word “blue” is shown in the color blue; and (c) incongruent: patients named the color of words in an incongruent font color, eg, the word “blue” is shown in the color red (Figure 1C). Each stimulus set under each condition was presented in two blocks of 24 trials, for a total of 48 trials, and the stimuli blocks were presented to the patient in a random order. For each trial, the subject was instructed to quickly press the color‐coded key that corresponded with the font color displayed on the computer screen. Colorblind patients were excluded from the study. The Stroop interference score (SI) was derived from the Maastricht Longitudinal study.^39^

$$\begin{array}{c} \text{Stroop Interference (SI) Score = Incongruent Reaction Time}\\ \;-\left(\frac{\text{Congruent Reaction Time}+\text{Neutral Reaction Time}}{2}\right)\\ \%\;\text{Accuracy}=100\%\\ \;\times \left(\frac{48\;\text{Total Responses}-\text{Number of Incorrect Responses}}{48\;\text{Total Responses}}\right) \end{array}$$

### 2.5 Statistical analysis

Descriptive analyses were used to describe demographic characteristics of the sample. Categorical variables such as ethnicity, T stage, Gleason scores, and androgen deprivation therapy usage were analyzed using the *chi*‐square test. Variables such as age, body mass index (BMI), and prostate‐specific antigen (PSA) levels were compared using the two‐tailed *t*‐tests. All data were expressed as mean ± SE of the mean. Normality of data distribution was examined using the Shapiro‐Wilk test. In cases when the test of homogeneity of variance was violated (significance < 0.05), the data were analyzed using nonparametric tests of significance. ANOVA was used for comparisons involving 3 groups or more. Pearson's correlation was used to assess association between two variables. A collinearity diagnostics procedure was performed prior to regression analysis to avoid overfitting and collinearity in constructing the multivariate model. In order to prevent spurious results, a tolerance value of greater than 0.4 was considered acceptable to exclude multicollinearity.^40^ ANCOVA with age, BMI, race/ethnicity, cancer T‐stage, and HAM‐D scores as covariates was performed to compare scores of specific items. *Post‐hoc* between‐group comparisons were performed using Mann‐Whitney *U* tests or *t* test depending on the normality of data distribution, with the Benjamini‐Hochberg false discovery rate correction for multiple comparisons. Statistical analyses were conducted using spss statistics software version 23 (IBM SPSS, Purchase).

## 3 RESULTS

### 3.1 Subject demographics

Subjects are men with non‐metastatic prostate cancer at an average age of 65.56 ± 7.22 years (Table 1). Of the total of 59 subjects, 24 subjects developed clinically meaningful fatigue (ΔFACT‐F ≥3) during RT. A minimum of 32 subjects would be needed to reach an alpha of 0.05 and a power of 0.8 based on a priori power analysis, indicating that the current study is sufficiently powered despite the small sample size. Fatigued and non‐fatigued groups did not differ in general clinical characteristics including age, BMI, T stage, Gleason scores, baseline PSA, and hemoglobin levels (Table 1). There was a small but not statistically significant difference in HAM‐D scores between the groups (fatigued: 1.08 ± 1.44, non‐fatigued: 2.26 ± 2.76, *P* = .06). However, neither group was considered clinically depressed as none of the HAM‐D scores was above 7.

**Table 1:** Demographics and clinical characteristics of sample population

	Total (N = 59)	Fatigued (n = 24)	Nonfatigued (n = 35)	*P* value
Age (y)	65.56 ± 7.22	67.21 ± 5.96	64.43 ± 7.86	.14
Height (cm)	174.72 ± 7.69	175.31 ± 8.03	174.31 ± 7.55	.63
Weight (kg)	91.08 ± 14.75	93.42 ± 15.00	89.48 ± 14.58	.32
BMI	29.79 ± 4.61	30.39 ± 4.89	29.39 ± 4.44	.42
Race/Ethnicity				
Asian	6.90%	8.33%	5.88%	.49
Black	32.76%	20.83%	41.18%	
Hispanic	3.45%	0.00%	5.88%	
White	56.90%	70.83%	47.06%	
Other	1.72%	0.00%	2.94%	
T‐stage				
T1c	48.94%	47.62%	50.00%	.91
T2a	23.40%	19.05%	26.92%	
T2b	4.26%	4.76%	3.85%	
T2c	6.38%	9.52%	3.85%	
T3a	10.64%	9.52%	11.54%	
T3b	6.38%	9.52%	3.85%	
Gleason score				
6	6.78%	8.33%	5.71%	.33
7	47.46%	50.00%	45.71%	
8	32.20%	20.83%	40.00%	
9	13.56%	20.83%	8.57%	
PSA (ng/mL)	13.08 ± 18.55	14.46 ± 20.38	12.14 ± 17.43	.64
ADT	71%	88%	60%	.02
Hemoglobin (g/dL)	13.24 ± 3.23	12.86 ± 2.87	13.50 ± 3.47	.46
HAM‐D	1.78 ± 2.38	1.08 ± 1.44	2.26 ± 2.76	.06

Note

Values are mean ± SD.

Abbreviations: ADT, androgen deprivation therapy; BMI, body mass index; HAM‐D, Hamilton Depression Rating Scale; PSA, prostate‐specific antigen; RT, radiation therapy.

### 3.2 Cognitive aspect of fatigue

**Figure 2:**
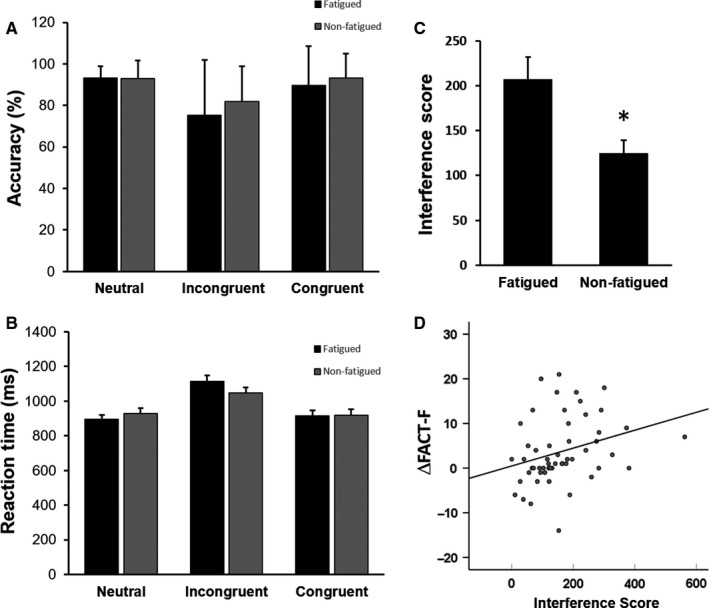
Cancer‐related fatigue is associated with altered cognitive interference but not performance accuracy. A, Fatigue did not affect Stroop performance accuracy in any of the three conditions (*F*_1156_ = 1.28, *P* = .26). B, Reaction time was significantly different among the three different conditions (*F*_2156_ = 16.85, *P* = 2.38 × 10^−7^). There was no interaction between fatigue and condition (*F*_2156_ = 0.89, *P* = .41). C, The Interference Score was significantly higher in the fatigued group (Fatigued 203.30 ± 26.00, nonfatigued 129.93 ± 15.14, *P* = .01). D, Fatigue also significantly correlated with the Stroop Interference Score (*r* = .389, *P* = .004). ​* indicates statistical significance *P* < .05.

**Figure 3:**
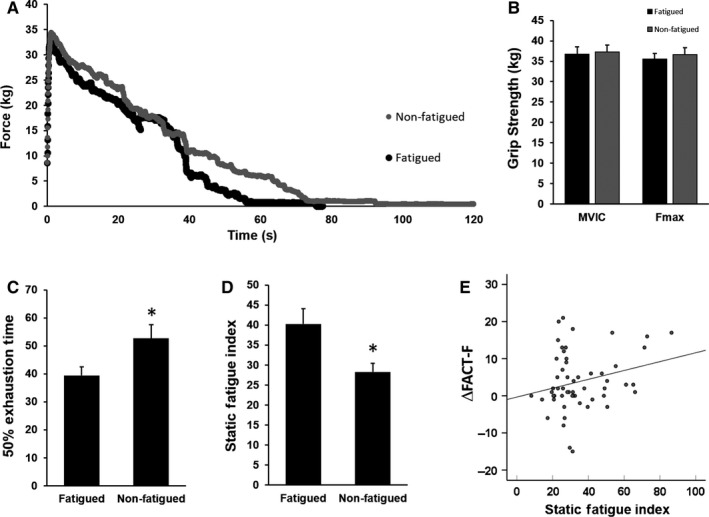
Cancer‐related fatigue is associated with physical fatigue. A, Mean hand grip strength of fatigued and nonfatigued subjects over time during the static fatigue test. B, No significant difference between fatigued and nonfatigued subjects was observed in maximal voluntary isometric contraction (MVIC; Fatigued 36.73 ± 1.76 kg, Nonfatigued 37.33 ± 1.68 kg, *P* = .81) or maximal force (*F*_max_) during the static fatigue test (Fatigued 35.50 ± 1.45 kg, Nonfatigued 36.65 ± 1.71 kg, *P* = .63). C, Fatigued subjects exhibited decreased 50% exhaustion time compared to nonfatigued subjects, indicative of decreased endurance (Fatigued 39.46 ± 3.08 s, Nonfatigued 52.68 ± 4.80 s, *P* = .03). D, Subject who reported subjective fatigue also exhibited higher static fatigue index compared to nonfatigued subjects (Fatigued 40.28 ± 3.80%, Nonfatigued 28.28 ± 2.15%, *P* = .01). E, ΔFACT‐F significantly correlated with the static fatigue index (*r* = .251, *P* = .03). ​* indicates statistical significance *P* < .05.

Fatigue did not affect the accuracy of performance on the Stroop test in any of the conditions tested (*F*_1156_ = 1.28, *P* = .26; Figure 3A). The average reaction time of the 48 trials was different among the 3 different conditions with the incongruent condition requiring the longest reaction time (F_2156_ = 16.85, *P* = 2.38 × 10^−7^; Figure 2B). However, there was no significant interaction between fatigue and stimuli condition (F_2156_ = 0.89, *P* = .41; Figure 2B). The SI score represents the reaction time difference in naming the color in the incongruent condition relative to the congruent and neutral conditions,^41^ which takes into account the baseline speed in color naming and word reading.^42^ Despite performing at the same level of accuracy, the fatigued group exhibited increased SI compared to nonfatigued subjects (*P* = .01; Figure 2C). Furthermore, the SI scores correlated significantly with ΔFACT‐F scores during RT, suggesting that subjective feeling of clinically meaningful fatigue was reflected in impaired cognitive interference control (*r* = .389, *P* = .004; Figure 2D).

### 3.3 Motor aspect of fatigue

The mean grip strength of both fatigued and nonfatigued patients during the static fatigue test is plotted as force (kg) over time (seconds; Figure 3A). We found no significant difference in MVIC in the maximum hand grip strength test (Figure 3B) between fatigued and nonfatigued subjects (Fatigued 36.73 ± 1.76 kg, Nonfatigued 37.33 ± 1.68 kg, *P* = .81). Further, no significant difference was observed in maximal hand grip strength (*F*_max_) during the static fatigue test (Fatigued 35.50 ± 1.45 kg, Nonfatigued 36.65 ± 1.71 kg, *P* = .63) between the two groups (Figure 3B). Maximum force measured in the maximum hand grip strength test (MVIC) and the static fatigue test (*F*_max_) in each subject differed by values ranging from 0.18% to 17.5% with a mean of 1.76%. The similarity between MVIC and *F*_max_ (*P* = .53; Figure 3B) indicates that sufficient effort was exerted during sustained contraction in the static fatigue test. Interestingly, 50% exhaustion time (50% ET), the time it took for maximal muscle contraction force to degrade to 50% of MVIC during static fatigue test, was significantly different between fatigued and nonfatigued subjects (Fatigued 39.46 ± 3.08 seconds, Nonfatigued 52.68 ± 4.80 seconds, *P* = .03; Figure 3C). The fatigued group also exhibited significantly higher static fatigue indices (SFI) (Fatigued 40.28 ± 3.80%, Nonfatigued 28.28 ± 2.15%, *P* = .01), indicative of a faster decline over time in maximal hand grip strength (Figure 3D). SFI significantly correlated with ΔFACT‐F (*r* = .25, *P* = .03; Figure 3E), suggesting a motor component of perceived fatigue experience.

## 4 DISCUSSION

Fatigue reported by cancer patients is characterized by reduced daily activity performance in both physical and cognitive domains.^3^, ^43^ Physical fatigue is more frequently reported possibly because it is easier for patients to notice and discuss compared to cognitive/psychological fatigue.^44^ This illustrates the limitations of using self‐report questionnaires when studying CRF. A strength of the current study is the combined use of the common self‐report questionnaires in conjunction with objective measures of the Stroop Color‐Word Interference Test to assess cognitive impairment, and a handheld dynamometer to measure physical fatigue. The main finding is that CRF was associated with both increased cognitive interference and decreased physical endurance, suggesting that the overall sense of tiredness reported by cancer patients is both cognitive and physical in nature.

Another major finding is that cognitive interference reaction time was significantly increased in fatigued subjects, performance accuracy was not affected. Furthermore, fatigued subjects exhibited increased motor fatigability both in terms of 50% ET and SFI, whereas maximal grip strength did not differ between the two groups. Our findings illustrate the importance of “time” as a variable in designing objective tests to measure CRF. For example, fatigued subjects required more time to overcome executive function interference, even though they performed at the same level of accuracy as nonfatigued subjects. In a similar sense, fatigued subjects had trouble sustaining maximal force over time, even though maximal grip force did not differ between the two groups. These findings suggest that objective measures designed to characterize CRF should consider including “time” as a factor. Fatigued subjects differed from nonfatigued subjects not in peak performance (eg, cognitive performance accuracy in the cognitive test, or maximal grip strength in the hand grip test), but in performance as a function of time (eg, time needed to override an overlearned cognitive function in the Stroop test, or time needed to sustain maximal grip force in the hand grip test).

Various questionnaires and scoring methods have been established to quantify CRF despite the lack of a consensus definition.^3^ The exact method used to clinically define fatigue influences outcomes in mechanistic investigations of fatigue.^25^ As patient‐reported outcomes tend to correlate poorly with the functional status, a more meaningful way to interpret the clinical relevance of self‐report questionnaire data is by using the MCID cutoff method.^24^ The MCID approach takes into account the subjectivity of self‐reported symptoms.^26^ A decrease of ≥3 points in FACT‐F scores reflects a meaningful change in the functional status of cancer patients and uses baseline scores as the internal reference point for each patient,^26^ and was selected in this study to characterize the cognitive and physical aspects of CRF.

A major challenge in objectively measuring physical fatigue is the lack of standardized tools.^31^ In terms of muscle fatigue, both the duration of contraction and indices calculated based on deterioration of strength over time have been demonstrated as useful tools.^45^ Previous studies have shown decreased endurance time and voluntary muscle recruitment in fatigued cancer patients.^46^ This is consistent with our finding that fatigued subjects exhibited shorter 50% ET and higher SFI, suggesting that physical fatigue was related to a more rapid decline in grip force over time. Cognitively, the utility of using the Stroop test to measure the ability to inhibit cognitive interference has been demonstrated in clinic and research settings.^35^ Subjective fatigue has been shown to correlate with the perception of increased cognitive effort.^47^ This supports our finding that even though performance accuracy was not different between the groups, the SI score was significantly higher in the fatigued group indicating increased cognitive effort spent to maintain task performance (Figure 2). Interestingly, fatigue in aging, multiple sclerosis, and chronic fatigue syndrome are also associated with an increase in reaction time but not performance accuracy.^48^ These studies along with our findings suggest that the cognitive aspect of fatigue, in the general sense, appears to be associated with increased cerebral activation across time, or prolonged effort, to maintain the same level of performance during tasks that require sustained mental effort.^10^, ^49^

Even though cognitive and motor fatigue may be distinguishable by symptom presentation, it is possible that they originate from the same upstream pathogenic mechanisms. Both cognitive and motor fatigue can be affected by inflammation associated with cancer or cancer treatment.^5^, ^14^, ^50^ Oxidative damage induced by RT can target sarcoplasmic reticulum calcium release channels affecting force generation capacity, as well as caspase/calpain pathways leading to contractile protein catabolism and muscle weakness.^51^, ^52^ In addition to directly influencing motor fatigability in the periphery, inflammatory mediators can cross the blood‐brain barrier resulting in secondary inflammation in the brain and cognitive impairment.^5^, ^53^, ^54^ In fact, prefrontal cortex dysfunction and executive function impairment have been observed in cancer patients,^55^ consistent with our finding that fatigued subjects exhibited higher interference scores, a process that requires executive inhibitory control. It is worth point out that the cognitive versus physical fatigue distinction is based on behavioral characterization and can originate from central and/or peripheral mechanisms. For example, perceived physical fatigue can be a result of oxidative damage to muscle tissues themselves leading to mitochondrial dysfunction and decreased ATP supplies, lactate buildup and intramuscular acidification, and K^+^ accumulation in the sarcolemma by high‐frequency depolarization.^56^, ^57^ The same sensation of physical fatigue can also arise from defects in the neuromuscular junctions or impaired descending drive from the motor cortex.^58^ Future studies will further examine whether physical fatigue in the prostate cancer cohort is related to central factors or peripheral muscular impairment. For example, M‐wave (muscle compound action potential) recorded using surface electromyography will help determine the contribution of the deterioration on sarcolemma excitability.^59^

A limitation of the study is that a small sample size of 59 subjects was included in the analyses. A priori power analysis determined that a minimum of 32 subjects would be needed to reach an alpha of 0.05 and a power of 0.8, indicating that the current study is sufficiently powered. While it is encouraging that these findings are robust enough to be detected in a relatively small sample size, future validation studies will be conducted with a larger sample size. Furthermore, we only included subjects with nonmetastatic prostate cancer receiving RT. Although the homogeneity of the sample allowed us to study CRF without influences from other clinical variables including the types of cancer and cancer treatment, additional investigations will be completed to examine whether these findings are generalizable to fatigue associated with other types of cancer and cancer treatment. In addition, the influence of other conditions (eg, cardiopulmonary, neurological, musculoskeletal conditions) and the use of concomitant medications (eg, antihypertensives, antilipidemic) should be investigate in future studies.

We previously showed that CRF during RT and one‐year post‐RT are dissociable in regard to comorbid symptoms and underlying mechanisms.^4^, ^60^ Future studies will focus on discerning the cognitive and physical contributions of different fatigue subtypes. As different tests may exhibit various sensitivity to the types of fatigue measured, future studies will expand on these findings and examine the association between CRF and performance on different objective measures.

In conclusion, we identified objective measures to assess cognitive and physical characteristics of CRF in cancer patients undergoing RT. We found that fatigued subjects needed more time to overcome cognitive interference in the domain of executive functioning. CRF also correlated with increased physical fatigability and an inability to sustain maximal grip strength over time. On the contrary, fatigue was not associated with peak performance in either cognitive performance accuracy or maximal grip strength. This suggests that perceived CRF based on self‐report manifests in both cognitive and physical domains as a function of time. To our knowledge, this is the first study that examines the contributions of both the cognitive and physical aspects of CRF in the same cohort. These findings provide new and important clues to advance our understanding of the CRF phenotype. Objective measures used in this study will pave the way for clinicians to accurately quantify various aspects of CRF. Further, these findings will help clinicians design more targeted and individualized therapeutic approaches to alleviate the socioeconomic burden of this debilitating disorder.

## CONFLICT OF INTEREST

None declared.

## AUTHOR CONTRIBUTION

All authors meet the criteria for authorship as defined by the ICMJE definition of authorship.
